# An unusual and severe case of paclitaxel‐induced hand‐foot syndrome

**DOI:** 10.1002/ccr3.3573

**Published:** 2020-11-29

**Authors:** Ichrak Ben Abdallah, Sonia Ben Nasr, Aref Zribi, Jihene Ayarai, Sana Fendri, Balti Mehdi, Haddaoui Abderrazek

**Affiliations:** ^1^ Department of medical oncology Faculty of medicine of Tunis Military Hospital of Tunis University Tunis El‐Manar Tunis Tunisia

**Keywords:** antineoplastic agents, hand‐foot syndrome, paclitaxel

## Abstract

Although paclitaxel is known to cause mild skin toxicity, it may induce severe HFS requiring drug withdrawal. Patients with high disease burden might receive prolonged paclitaxel chemotherapy. Hence, a grade 2 toxicity would better indicate withdrawal of paclitaxel instead of suspension and rechallenge, to prevent such severe HFS requiring long‐time recovery.

## Graphical abstract

Although paclitaxel is known to cause mild skin toxicity, it may induce severe HFS requiring drug withdrawal. Patients with high disease burden might receive prolonged paclitaxel chemotherapy. Therefore, a grade 2 toxicity would better indicate withdrawal of paclitaxel instead of temporary suspension to prevent such severe HFS requiring long‐time recovery.

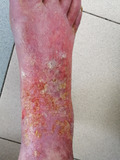

## 1 COMMENT

**FIGURE 1:**
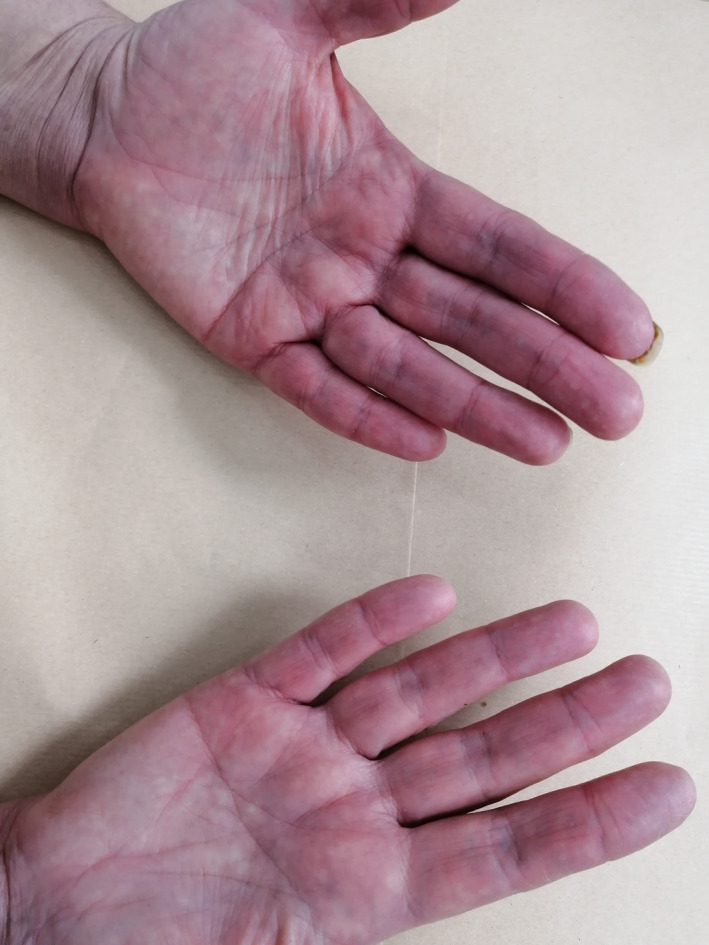
Erythematous and violaceous lesions induced by paclitaxel

**FIGURE 2:**
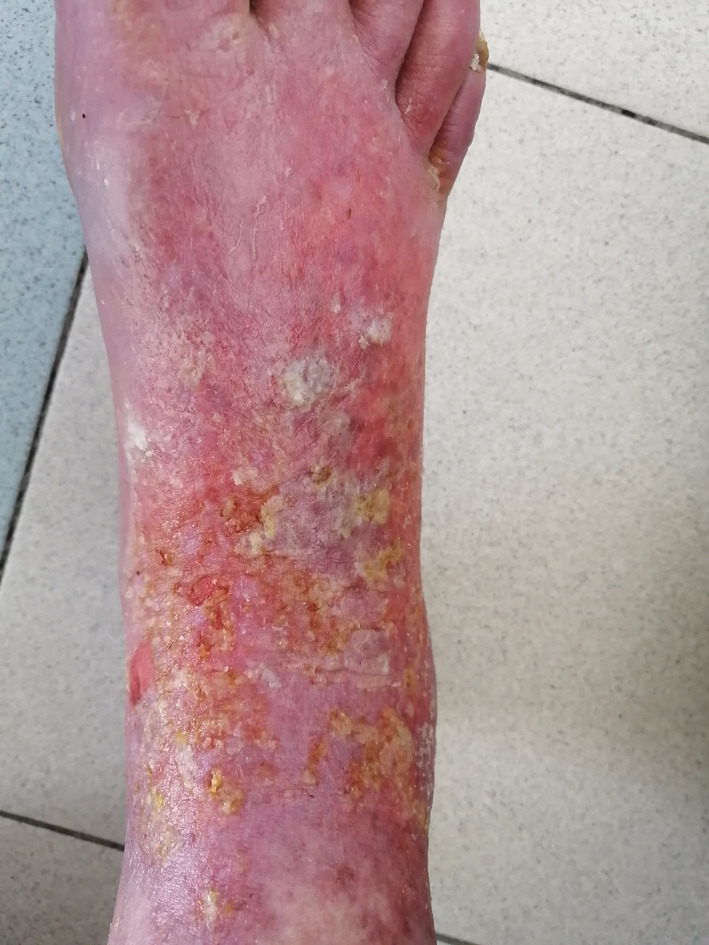
Paclitaxel‐induced ulcerations and epidermal necrosis of the dorsum of the foot

**FIGURE 3:**
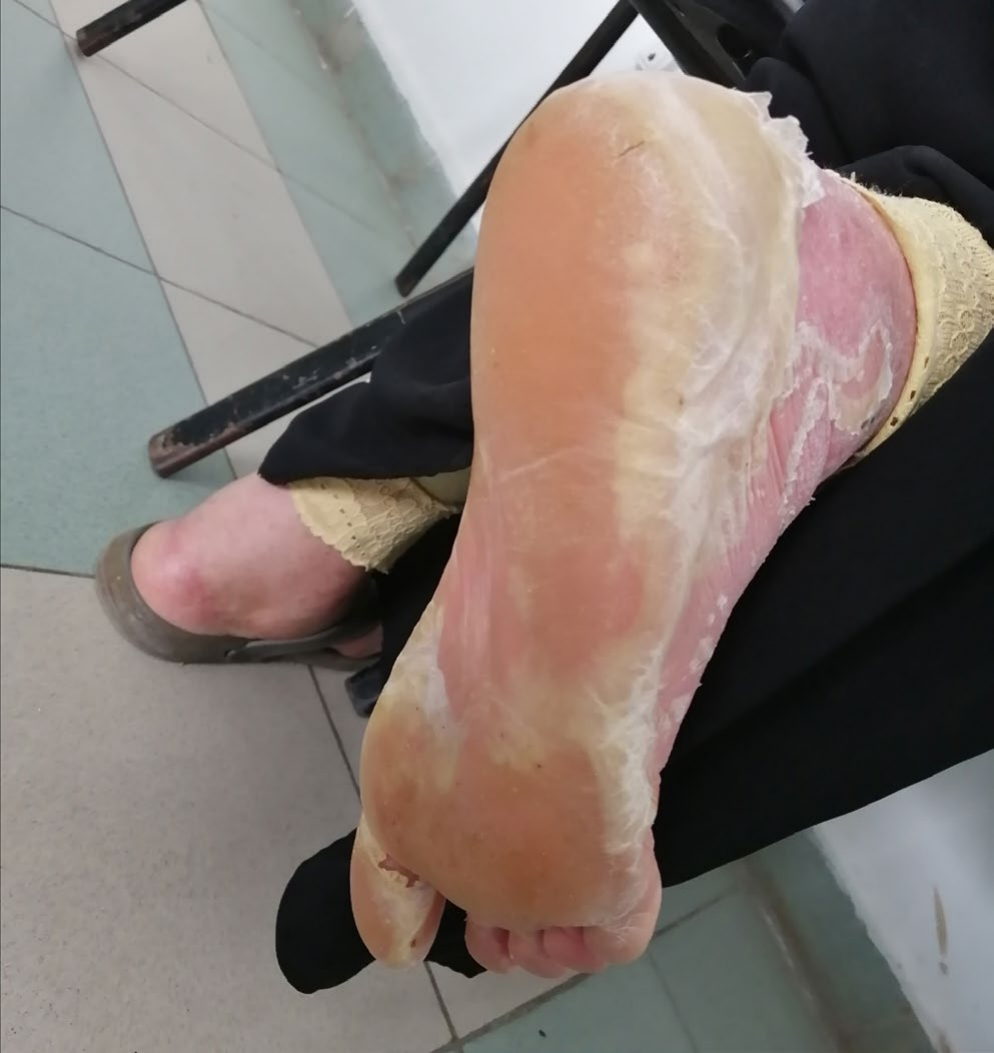
Paclitaxel‐induced severe peeling feet

**FIGURE 4:**
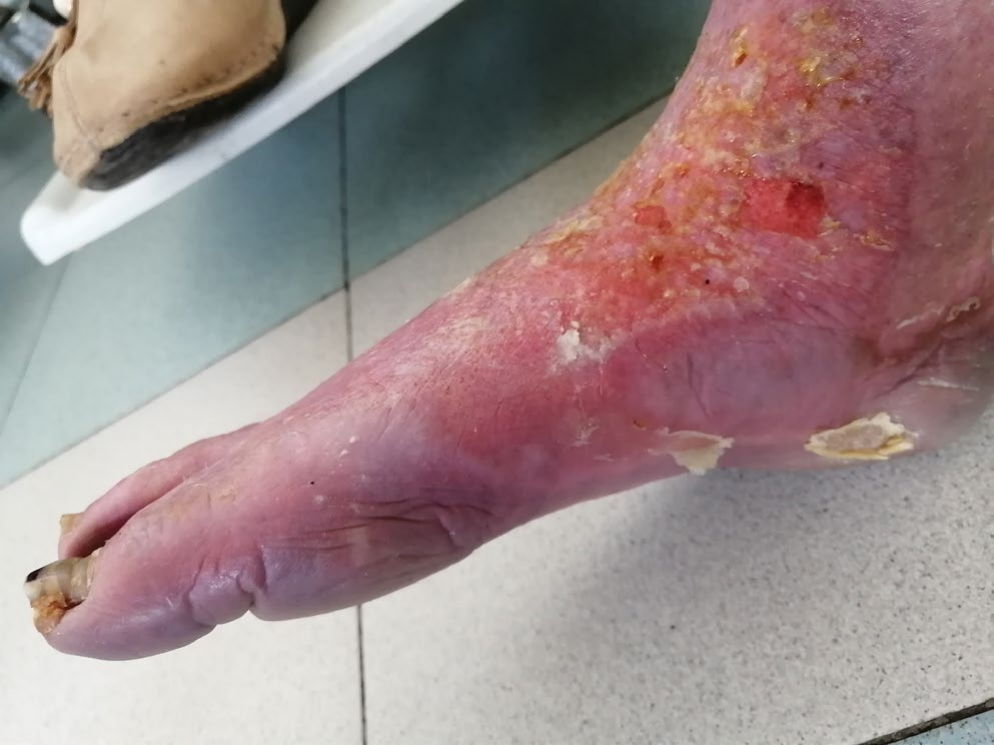
Paclitaxel‐induced peeling, bleeding, and onycholysis

Although paclitaxel is known to induce skin adverse events, acral erythema has been described only in few case reports.^1^ We herein report the case of a 44‐year‐old premenopausal female patient who was referred to our department for metastatic breast cancer. She had a history of acute coronary syndrome 9 months earlier receiving bisprolol 2.5 mg daily, aspirin, and atorvastatin. The pathology report showed mixed ductal‐lobular invasive carcinoma, HER2 negative, hormone receptor‐positive. Staging workup revealed multiple metastases in the liver with diffuse peritoneal carcinosis. As anthracyclines were contraindicated, first‐line weekly paclitaxel at a dose of 80 mg/m^2^ was initiated. Over nine courses, the patient had a complete peritoneal response and partial response to hepatic lesions. Main toxicities were mild ungueal toxicity and alopecia. Maintenance therapy with letrozole was carried out for 4 months. The patient presented then liver disease progression with abundant ascites. Weekly paclitaxel was reintroduced at the same dose with premedication before each infusion (corticosteroids, anti‐H1, and setrons). After six infusions, the patient presented WHO grade 2, hand‐foot syndrome, which resolved 2 weeks after chemotherapy discontinuation. Paclitaxel was reintroduced with preventive topical emollients and a 20% dose reduction. After three other courses, the patient presented erythematous and violaceous lesions of her palms (Figure 1) and multiple ulcerations and epidermal necrolysis of the dorsum of her foot (Figure 2) associated with severe peeling soles and bleeding (Figures 3 and 4). The patient was referred to the dermatology department, where analgesics, emollients, topical steroids, and antibiotics were prescribed. Paclitaxel was then withdrawn. Three weeks later, the patient died due to disease progression, as no other chemotherapy could be initiated.

## CONFLICT OF INTEREST

None declared.

## AUTHORS' CONTRIBUTION

IBA: collected the patient's data and drafted the manuscript. SBN, AZ, JA, and SF: revised the manuscript and gave critical review of the content. AH and MB: gave approval for the final version to be published after review.

## ETHICAL APPROVAL

The patient gave verbal and written consent for the publication of these clinical images.

## Data Availability

The patient's records are available from the corresponding author.
